# Incidence, Predictors and Outcomes of Noninvasive Ventilation Failure in Very Preterm Infants

**DOI:** 10.3390/children9030426

**Published:** 2022-03-17

**Authors:** Sara M. Fernandez-Gonzalez, Andrea Sucasas Alonso, Alicia Ogando Martinez, Alejandro Avila-Alvarez

**Affiliations:** 1 Neonatology Department, Complexo Hospitalario Universitario A Coruña (CHUAC), 15006 A Coruña, Spain; sara.maria.fernandez.gonzalez@sergas.es (S.M.F.-G.); andrea.sucasas.alonso@sergas.es (A.S.A.); alicia.ogando.martinez@sergas.es (A.O.M.); 2 A Coruña Biomedical Research Institute (INIBIC), 15006 A Coruña, Spain

**Keywords:** continuous positive airway pressure, hyaline membrane disease, noninvasive ventilation, prematurity and respiratory distress syndrome

## Abstract

Non-invasive ventilation (NIV) is now considered the first-line treatment for respiratory distress syndrome in preterm infants. We aimed to evaluate the rates of non-invasive ventilation failure rate in very preterm infants, as well as to identify its predictors and associated outcomes. We designed a single-center retrospective cohort study including infants ≤32 weeks gestational age and ≤1500 g. The NIV failure was defined as the need for intubation at <72 h of life. After applying inclusion and exclusion criteria, 154 patients were included in the study, with a mean GA of 29.7 ± two weeks. The NIV failure rate was 16.2% (*n* = 25) and it was associated with lower bronchopulmonary dysplasia (BPD)-free survival (OR 0.08; 95% CI 0.02–0.32) and higher incidence of intraventricular hemorrhage > II (OR 6.22; 95% CI 1.36–28.3). These infants were significantly smaller in GA and weight. Higher FiO_2_ during resuscitation (OR 1.14; 95% CI 1.06–1.22) and after surfactant administration (OR 1.17; 95% CI 1.05–1.31) represented independent risk factors for NIV failure. In conclusion, NIV failure is frequent and it could be predicted by a higher oxygen requirement during resuscitation and a modest response to surfactant therapy. Importantly, this NIV failure is associated with worse clinical outcomes.

## 1. Introduction

Respiratory distress syndrome (RDS) remains the most common respiratory cause of morbidity and mortality in preterm infants. The incidence of RDS increases with decreasing gestational age (GA), affecting the vast majority of infants <28 weeks GA and up to 30% of infants in the range of 28 to 34 weeks GA [1].

The approach to RDS has evolved during the last decades. Landmark randomized clinical trials demonstrated that nasal continuous positive airway pressure (CPAP) was an effective and safe alternative to endotracheal intubation in the management of very preterm infants with RDS [2,3]. Moreover, invasive mechanical ventilation (IMV) was shown to be associated with ventilator-induced injury and bronchopulmonary dysplasia (BPD) [4]. Therefore, in current neonatal practice, a wide consensus exists that avoidance of IMV when possible is of benefit. In fact, both current American [5] and European Guidelines [6] favor the use of CPAP in spontaneously breathing preterm babies rather than intubation.

In keeping with these recommendations, a number of studies have recently shown an increase in the use of non-invasive ventilation (NIV) strategies in neonatal units worldwide [7].

CPAP, as well as other types of NIV, are part of current protocols aiming for lung protection, which also include antenatal steroid administration and early selective surfactant therapy. Its inclusion in clinical practice has led to the avoidance of IMV in the majority of very preterm infants [1,8]. However, how these changes in the initial approach translate into better respiratory outcomes later on is less clear, and there is still a significant group of infants in which noninvasive strategy fails and finally require tracheal intubation due to evolving RDS [2].

This NIV failure may be associated with worse outcomes [2,9] and, therefore, its prediction and early detection remains a challenge in neonatal medicine [10,11]. A more accurate characterization of RDS and a personalized approach to its treatment has been recently advocated [12]. In our opinion, detailed study of NIV failure should be the basis of this personalized approach and for the assessment of new prognostic and therapeutic tools.

Therefore, we designed a retrospective observational study with the main objective of describing the NIV failure rate in preterm infants ≤32 weeks GA, as well as identifying its predictors and associated outcomes. Our hypothesis was that NIV failure is still prevalent and that some bedside clinical variables may predict its occurrence.

## 2. Materials and Methods

This was a retrospective cohort study of prospectively collected data carried out in a tertiary neonatal intensive care unit (NICU) of the Spanish national health service. We collected data of preterm infants ≤32 weeks GA and ≤1500 g who were admitted to the NICU between 1 January 2015 and 30 August 2020 for active support. Since our study focused on NIV approach, infants requiring intubation in the delivery room (DR) or in the first hour of life, as well as infants who did not require any type of respiratory support, were excluded from this analysis. Life-threatening malformations or chromosomal anomalies were also exclusion criteria. The study was approved by the local Clinical Research Ethics Committee and parental written consent was obtained before inclusion.

### 2.1. Study Outcomes and Variables

The main outcome of our study was NIV failure, defined as the need for intubation within 72 h of life. Based on this outcome, the study sample was divided into two different groups: patients in whom NIV was successfully applied constituted the ‘noninvasive success group’ (NSG) and those who required intubation within 72 h of life constituted the ‘noninvasive failure group’ (NFG).

The following variables were recorded and compared between groups: demographic data at birth and at screening (GA, birth weight [BW], sex, Apgar score at 1 and 5 min, temperature at admission); perinatal characteristics (antenatal steroids, maternal hypertension, chorioamnionitis, in vitro fertilization, mode of delivery); respiratory support (IMV, Non-invasive positive pressure ventilation [NIPPV], surfactant, FiO_2_ at different times); respiratory outcomes (BPD, pneumothorax, days on IMV, days on supplementary oxygen) and other relevant clinical outcomes (necrotizing enterocolitis [NEC], patent ductus arteriosus, intraventricular hemorrhage [IVH], retinopathy of prematurity, spontaneous intestinal perforation, mortality, hospital stay). BPD was defined as the need for supplementary oxygen for at least 28 days and classified as moderate or severe depending on oxygen requirements and ventilator support at 36 weeks postmenstrual age (moderate if supplementary oxygen only, and severe if respiratory support) [11,13]. Only necrotizing enterocolitis ≥ grade 2, as defined by Bell et al. [14], was considered. Patent ductus arteriosus was diagnosed by cardiac ultrasound and managed according to local protocols (only ductus >1.5 mm were considered). Intraventricular hemorrhage was defined and graded according to Volpe [15]. All infants were screened for retinopathy of prematurity according to national guidelines [16]. Small for gestational age (SGA) was defined as a birth weight zscore < −1.5 according to 2013 Fenton growth charts.

### 2.2. Respiratory Management

There were standard guidelines for respiratory management in place in the NICU during the study period, which were in keeping with European and national recommendations [6,17]. In brief, stabilization in the DR was initiated with mask and T-resuscitator applying CPAP or NIPPV depending on the presence of spontaneous breathing and heart rate. NIPPV was used in the NICU as early rescue therapy before considering orotracheal intubation or as initial support at clinical discretion. All types of NIPPV (BiPAP devices or conventional ventilators) were used in the unit. Surfactant was administered using the INSURE (intubation-surfactant-extubation) procedure in the first hours of life when oxygen requirements were greater than 30% or if the infant required orotracheal intubation for any other reason. LISA was not in place in the unit during the study period. For the INSURE technique, premedication with caffeine, fentanyl and atropine was administered. For intubation and connection to IMV premedication with fentanyl, rocuronium and atropine was administered.

Caffeine was given prophylactically to all infants <28 weeks GA and in those <32 weeks who developed apneas (20 mg/Kg bolus followed by 5 mg/Kg per day, which can be increased to 10 mg/Kg if clinically needed). Hydrocortisone administration was considered in infants on IMV and FiO_2_ > 0.3 after the first week of life. The local hydrocortisone regimen lasts 22 days (total dose 72.5 mg/kg) [18].

### 2.3. Statistical Analysis

Descriptive data were presented as mean ± standard deviation (SD) for normally distributed variables and as median (interquartilic range) for non-normal, or *n* (%) for qualitative variables. Differences between NFG and NSG were first explored by univariate analysis. Categorical variables were analyzed by χ^2^ and Fisher’s exact tests and continuous variables were assessed by Student’s *t*-test. Logistic regression models were then performed for multivariate analysis adjusting by GA (model 1) and by GA, sex, SGA and postnatal steroids (model 2). Unadjusted and adjusted odds ratios (OR) and their 95% confidence intervals were calculated for selected variables. *p*-value less than 0.05 was considered statistically significant. Analyses were performed using IBM SPSS statistical software for Windows v. 24.0.

## 3. Results

**Figure 1:**
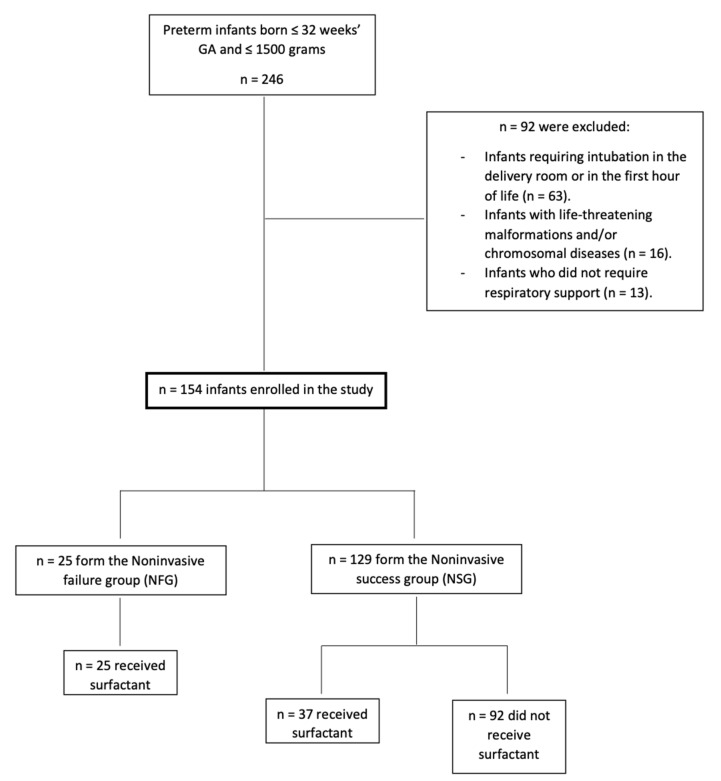
Flow chart depicting recruitment of cohort.

From January 2015 to August 2020, a total of 246 infants ≤32 weeks GA and ≤1500 g were admitted to the study NICU. After applying exclusion criteria (see flow chart, Figure 1) a total of 154 newborns (49.4% girls) were finally included in the study. The mean GA and birth weight of the entire cohort were 29.79 ± 2 weeks and 1153.6 ± 260 g, respectively.

Among the patients included in the study, 25 (16.2%) required orotracheal intubation and IMV within the first 72 h of life and constituted the NFG. A total of 62 patients (40.3%) received surfactant treatment at a mean age of 5.24 ± 9.16 h. Apart from the NFG, another 10 patients were intubated at some time during NICU admission.

Some demographic and clinical features were significantly different between the NFG and NSG. As shown in Table 1, patients in the NFG were smaller (28.22 ± 2.33 vs. 30.09 ± 1.79 weeks GA; *p* <0.001) and required higher FiO_2_ during DR stabilization (31.61 ± 6.52 vs. 52.56 ± 23.79; *p* <0.001). A FiO_2_ cutoff of 53% provided a sensitivity = 98%, a specificity of 64%, a positive predictive value = 90% and a negative predictive value = 83%. Among patients that received surfactant (*n* = 62), FiO_2_ after surfactant administration was also higher in the NFG (24.35 ± 5.01 vs 33.52 ± 12.27; *p* = 0.003).

Regarding outcomes, patients in the NFG showed a worse clinical course than patients in the NSG. Specifically, NFG infants showed significantly higher mortality rate, higher incidence of BPD, severe IVH and domiciliary oxygen. NICU stay and total duration of supplementary oxygen were also longer in the NFG. Other outcomes did not reach statistical significance but trends were always in the direction of worse clinical course in the NFG. Please refer to Table 2 for numerical data and further details.

After adjustment by confounders (Table 3), maximum FiO_2_ during DR stabilization and after surfactant treatment remained associated with NIV failure (OR 1.15; CI95% 1.07–1.24 and 1.17; CI95% 1.05–1.30, respectively). NIV failure was also associated with lower rates of survival without BPD (OR 0.08; CI95% 0.02–0.32) and survival without moderate-to-severe BPD (OR 0.02; CI95% 0.004–0.11), as well as higher rates of pneumothorax and severe IVH (OR 17.8; CI95% 1.65–192.9 and 6.22 CI95% 1.36–28.3, respectively).

## 4. Discussion

This study, which was conducted in a population of very preterm infants managed in the same neonatal unit, showed that a significant proportion of neonates still fail on NIV support and, most importantly, that those infants failing the NIV approach had significantly worse clinical outcomes.

NIV is now considered first-line treatment for RDS in preterm infants [6]. In our cohort of 246 preterm neonates ≤32 weeks GA at birth, almost 70% received NIV as initial respiratory support, but up to 16% of these infants finally required orotracheal intubation and mechanical ventilation early in the course of RDS. Our results are in keeping with previously reported NIV failure rates, which widely range from 13 to 50%. Roberts et al. [3] recently published a secondary analysis of data from a randomized trial focusing on NIV failure. They showed a NIV failure rate of 13.5%, slightly lower than ours, but with a different study population (28–36 vs. <32 weeks GA). Other studies included smaller babies and reported failure rates by GA. Dargaville et al. [2] showed a NIV failure rate of 43% among infants between 25–28 GA, which decreased to 21% in those infants between 29–32 weeks GA. Similarly, Kakkilaya et al. [19] reported a failure rate close to 50% in a population with lower mean GA and lower rate of ANS than ours. 

Unsurprisingly, and in keeping with these studies, our data shows that the smaller the infant, the higher the NIV failure rate. In our study, preterm infants under 28 weeks of GA have a failure rate as high as 64%, in comparison with 8.5% in the group of infants >28 weeks of GA. Therefore, as it was previously reported, low GA is the most important risk factor for NIV failure [3,9]. However, the clinical challenge at the bedside remains, at a given GA, how to reliably predict from data that are readily available which infants will require intubation.

Recently, De Luca and co-authors advocated for a more accurate characterization and a personalized approach to RDS [12]. In this scenario, a detailed study of risk factors associated with NIV failure may be the basis for any quality improvement or innovative proposal. Roberts et al. [3] showed that, apart from a lower GA, higher FiO_2_ requirements predicted the need for intubation within 72 h. This is a common finding in most of the studies focusing on prediction of intubation or surfactant requirement [3,9,10,19] and constitutes the rationale for the indication of surfactant based on FiO_2_ [6]. In our cohort, NIV failure can be predicted by oxygen requirements during DR stabilization and after surfactant therapy. We should acknowledge that FiO_2_ alone has many limitations as a tool to evaluate the oxygenation status, as it is highly influenced by factors other than RDS severity or oxygenation itself (i.e., oxygen targets, hemoglobin, temperature, peripheral perfusion, etc.). However, it is simple and readily available information that can at least be used to stratify patients according to their risk for NIV failure while novel tools are increasingly incorporated into clinical practice (lung ultrasound or advanced oxygenation metrics) or developed in basic research (surfactant biological tests).

FiO_2_ requirement after surfactant replacement therapy reserves further discussion. Surfactant is now used as an early treatment in patients with evolving RDS and unfavorable course on NIV. Therefore, in our study all of the patients in the NFG and 37 (28.7%) in the NSG received surfactant. In these patients, the FiO_2_ required after surfactant administration was independently associated with NFG. In fact, FiO_2_ dropped seven points in the NSG after surfactant, while it dropped only three points in the NFG. These findings may be an indication of the clinical response to surfactant and could be considered as a predictor of unfavorable respiratory course in clinical practice, which could serve as an early signal for intensify treatments, such as increasing the NIV settings.

Apart from the prediction of NIV failure, the other important question at the bedside for both clinicians and families have to do with the consequences of NIV failure when NIV is used as first-line respiratory support. Previous studies have shown that this failure is associated with adverse outcomes including death, BPD, IVH, and longer duration of IMV and hospitalization [2,3,9,20]. These findings were replicated in our study, in which infants in the NFG showed a significantly worse clinical course. The lower survival without BPD rates, as well as the higher need for home supplemental oxygen and the higher severe IVH rates may have a significant impact on long-term outcomes, quality of life, and resource consumption [21,22].

There are several explanations for our findings. Invasive endotracheal tube ventilation is considered the main risk factor for BPD and also negatively influences other outcomes [4,23]. Hence, consequences of NIV failure may be simply explained by the effect of IMV itself. Moreover, one common concern of clinicians facing a preterm infant with RDS is not to go one step behind his or her clinical course, as a delay in surfactant treatment is also associated with worse outcomes. In our cohort, 32% of NFG infants received surfactant after 3 h of life and mean age for surfactant administration in this group was 3.72 ± 4.43, which although similar to other series [20], may be less effective than earlier administration. Finally, given our study design and although we tried to adjust by confounders, we cannot rule out that individual differences related both with outcomes and NIV failure were present.

A striking morbidity that resulted in association with NIV failure in our cohort was spontaneous intestinal perforation (0% vs. 12%, *p* = 0.004). Conversely, previous studies did not describe significant differences in this outcome [3], and we speculate if the higher rates of NIPPV or hydrocortisone treatment in the NFG group may have influenced our results [24]. Unfortunately, the lack of precise data regarding timing and clinical presentation of spontaneous intestinal perforation in our database preclude a deeper analysis.

There are some limitations in this study. Its single-center retrospective nature may limit generalization of results. Moreover, observational studies have inherent biases that should prompt a cautious interpretation of conclusions, since causality cannot always be assumed. We cannot exclude the notion that other changes in the global management of infants during the study period may have influenced our results. The lack of a standardized protocol for the use of NIPPV devices is another limitation of our study, as well as a mean GA in the study sample older than other studies. However, we strongly believe that our findings are important as hypothesis generating and can serve as the basis for further study of NIV failure.

## 5. Conclusions

In conclusion, NIV failure among very preterm infants is relatively frequent and the difficulty of judging which infant is at higher risk continues. However, it seems that NIV failure can be predicted by a higher oxygen requirement in the DR and a modest response to surfactant therapy. Importantly, when intubation occurs, it is independently associated with a lower survival without BPD and a higher risk of IVH or pneumothorax. In our opinion, strategies to avoid NIV failure and to help clinicians to better predict which infants will fail on NIV should be strongly promoted.

## Figures and Tables

**Table 1:** Demographic and baseline variables.

$\underline{X}\;$ **± DS** **....**	**Total** ***n* = 154**	**NSG** ***n* = 129**	**NFG** ***n* = 25**	***p*** **-Value**
**Gestational age** **Birth weight** Temperature at admission Apgar score at 1 min Apgar score at 5 min	29.79 ± 2.00	30.09 ± 1.79	28.22 ± 2.33	**0.001**
	1185 (960–1410)	1200 (975–1420)	1000 (860–1245)	**0.003**
	35.86 ± 0.64	35.85 ± 0.61	35.92 ± 0.82	0.616
	6.97 ± 1.27	6.95 ± 1.18	7.04 ± 1.70	0.805
	8.29 ± 0.97	8.25 ± 0.93	8.48 ± 1.12	0.280
**Maximum FiO_2_ during DR stabilization**	36.41 ± 15.39	31.61 ± 6.52	52.56 ± 23.79	**<0.001**
Age at surfactant administration (hours) *n* = 62	5.24 ± 9.16	6.27 ± 11.25	3.72 ± 4.43	0.287
FiO_2_ before surfactant administration *n* = 62	33.49 ± 7.80	31.72 ± 6.08	36.33 ± 9.47	0.077
**FiO_2_ after surfactant *n* = 62**	27.67 ± 9.41	24.35 ± 5.01	33.52 ± 12.27	**0.003**
FiO_2_ decrease after surfactant *n* = 62	5.47 ± 7.37	7.13 ± 4.73	2.64 ± 10	0.097
***n* (%)**	**Total** ***n* = 154**	**NSG** ***n* = 129**	**NFG** ***n* = 25**	***p*** **-Value**
**GA ≤ 28**	48 (31,2)	32 (24,8)	16 (64)	**<0.001**
IVF	44 (28.6)	35 (27.1)	9 (36)	0.468
PIH	41 (26.6)	34 (26.4)	7 (28)	1
Female infant	76 (49.4)	66 (51.2)	10 (40)	0.384
ANS	153 (99.4)	128 (99.2)	25 (100)	1
Multiple birth	59 (38.3)	47 (36.4)	12 (48)	0.369
Cesarean section	120 (77.9)	101 (78.3)	19 (76)	1
SGA	17 (11)	14 (10,9)	3 (12)	0.867
Chorioamnionitis	23 (14.9)	17 (13.2)	6 (24)	0.220
**Surfactant**	62 (40.3)	37 (28.7)	25 (100)	**<0.001**
**NIPPV as initial support**	52 (33.8)	38 (29.5)	14 (56)	**0.032**
**Caffeine**	145 (94.2)	126 (97.7)	19 (76)	**<0.001**

Bolded values denote *p* < 0.05; Quantitative variables are presented as mean ± standard deviation (SD) for normally distributed variables and as median (interquartilic range) for non-normal ones. NSG, non-invasive success group; NFG, non-invasive failure group; FiO_2_, fraction of inspired oxygen; DR, delivery room; IVF, in vitro fertilization; PIH, pregnancy-induced hypertension; ANS, antenatal steroids; SGA, small for gestational age; NIPPV, non-invasive intermittent positive ventilation.

**Table 2:** Outcome variables.

***n* (%)**	**Total** ***n* = 154**	**NSG** ***n* = 129**	**NFG** ***n* = 25**	***p*-Value**
**Died**	10 (6.5)	0 (0)	10 (40)	**<0.001**
**BPD**	24 (15.6)	16 (12.4)	8 (32)	**0.022**
**BPD moderate-to-severe**	7 (4.5)	3 (2.3)	4 (16)	**0.014**
**Survival without BPD**	120 (77.9)	113 (87.6)	7 (28)	**<0.001**
**Survival without moderate-to-severe BPD**	137 (89)	126 (97.7)	11 (44)	**<0.001**
**Home supplementary oxygen**	10 (6.5)	1 (0.8)	9 (36)	**<0.001**
**IMV during admission**	35 (22.7)	11 (8.5)	25 (100)	**<0.001**
**Steroids for BPD**	11 (7.1)	4 (3.1)	7 (28)	**<0.001**
**Pneumothorax**	5 (3.2)	1 (0.8)	4 (16)	**0.002**
**IVH > II**	11 (7.1)	4 (3.1)	7 (28)	**<0.001**
PVL	6 (3.9)	4 (3.1)	2 (8)	0.250
ROP > II	5 (3.8)	3 (2.5)	2 (13.3)	0.097
PDA	22 (14.3)	17 (13.2)	5 (20)	0.532
Surgery for PDA	6 (3.9)	4 (3.1)	2 (8)	0.251
Ibuprofen for PDA	13 (8.4)	10 (7.8)	3 (12)	0.445
NEC (≥ stage 2)	7 (4.5)	5 (3.9)	2 (8)	0.610
**Isolated intestinal perforation**	3 (1.9)	0 (0)	3 (12)	**0.004**
Culture positive sepsis	46 (29.9)	38 (29.5)	8 (32)	0.810
$\underline{X\;}$± **DS**	**Total** ***n* = 154**	**NSG** ***n* = 129**	**NFG** ***n* = 25**	***p*-Value**
**Weight at discharge**	2256.21 ± 436.36	2313.13 ± 319.23	1925 ± 774.64	**0.033**
Weight Z-Score at discharge	−1.49 ± 1.15	−1.50 ± 1.19	−1.38 ± 0.88	0.652
Age at first intubation (hours) *n* = 35	42.85 ± 83.35	210 ± 124.52	13.78 ± 11.54	0.051
Duration of hospitalization (days) *	57.51 ± 63.57	56.91 ± 66.84	62.60 ± 21.35	0.744
NICU length of stay (days) *	21.79 ± 14.34	20.84 ± 13.45	29.80 ± 19.20	**0.022**
MV (hours) *	96 (34–216)	72(36–168)	96 (31–306)	0.221
NIV (hours) *	96 (48v168)	96 (48–168)	72 (48–216)	0.210
Supplemental oxygen (hours) *	192 (68–648)	192 (72–564)	120 (65–972)	**0.009**
**Number of doses of surfactant**	1 (1–1.25)	1 (1–1)	1 (1–2)	**0.003**

Bolded values denote *p* < 0.05; * Only among survivors; Quantitative variables are presented as mean ± standard deviation (SD) for normally distributed variables and as median (interquartilic range) for non-normal ones. BPD, bronchopulmonary dysplasia; IVH, intraventricular hemorrhage; PVL, periventricular leukomalacia; ROP, retinopathy of prematurity; PDA, patent ductus arteriosus; NEC, necrotizing enterocolitis; NICU, neonatal intensive care unit; MV, mechanical ventilation; NIV, non-invasive ventilation.

**Table 3:** Odds ratios and their 95% confidence intervals for selected variables.

	**Unadjusted**		**Adjusted by GA**		Adjusted by GA, SGA, Sex, Postnatal Steroids **	
	**OR**	**CI95%**	**OR**	**CI95%**	**OR**	**CI95%**
Birth weight *	0.78	0.66–0.93	1.03	0.80–1.32	0.97	0.69–1.37
**NIPPV as initial support**	2.83	1.16–6.87	1.03	0.34–3.06	0.97	0.32–2.92
**Maximum FiO_2_ during DR stabilization**	1.15	1.08–1.23	1.14	1.06–1.22	1.15	1.07–1.24
**FiO_2_ after surfactant**	1.16	1.04–1.29	1.17	1.05–1.31	1.17	1.05–1.30
**Outcomes**						
**BPD**	3.32	1.23–8.94	1.49	0.49–4.55	0.53	0.10–2.74
**Moderate-to-severe BPD**	8.00	1.67–38.3	6.07	1.07–34.4	3.77	0.61–23.1
**Survival without BPD**	0.05	0.02–0.15	0.07	0.02–0.25	0.08	0.02–0.32
**Survival without moderate-to-severe BPD**	0.01	0.00–0.07	0.02	0.00–0.12	0.02	0.00–0.11
**Home supplementary oxygen**	72.0	8.55–606.0	37.7	4.22–337.2	96.7	8.26–1131.5
**Pneumothorax**	24.3	2.59–228.8	20.2	2.01–204.7	17.8	1.65–192.9
**IVH > II**	12.1	3.23–45.6	5.43	1.31–22.4	6.22	1.36–28.3

* every 100 g; ** Adjusted by postnatal steroids only for respiratory outcomes; OR, odds ratio; GA, gestational age; SGA, small for gestational age; BPD, survival without BPD and home supplementary oxygen); NIPPV, non-invasive intermittent positive ventilation; BPD, bronchopulmonary dysplasia; FiO_2_, fraction of inspired oxygen; IVH, intraventricular hemorrhage.

## Data Availability

All of the data will be available on reasonable request following publication.
